# Contraceptive consultations: A cross-sectional study of Norwegian women's experiences and opinions

**DOI:** 10.18332/ejm/109773

**Published:** 2019-06-26

**Authors:** Kristin E. Forsberg, Ragnhild Lassemo, Mirjam Lukasse

**Affiliations:** 1 Department of Nursing and Health Sciences, Faculty of Health and Social Sciences, University of South-Eastern Norway, Kongsberg, Norway; 2 Akershus University Hospital, Nordbyhagen, Norway; 3 Institute of Nursing and Health Promotion, Faculty of Health Sciences, Oslo Metropolitan University, Oslo, Norway

**Keywords:** midwife, hormonal contraceptives, side effects, contraceptive consultations, sexual wellbeing

## Abstract

**INTRODUCTION:**

Access to contraceptive consultations and the content of these consultations are important to achieve a safe and satisfying sexual life and successful reproduction when desired. The aim of this study was to investigate Norwegian women’s experiences of and opinions on contraceptive consultations.

**METHODS:**

We conducted a cross-sectional study with a questionnaire distributed via Facebook with 1917 respondents of age ≥15 years. Descriptive analyses were used.

**RESULTS:**

Few women found it hard to access consultations (5%). Across all age groups, side effects were the most common topic that women (69%) wanted more information about. Concern about side effects was also the most frequent reason given (27%) for not using hormonal contraceptives. Among women aged 25–34 years, 54% wanted to know more about different available contraceptives. The majority of women deemed issues of sexual wellbeing important to address during a contraceptive consultation. Few women reported that these topics had been raised. Just under half of the women found it appropriate to see a midwife for contraceptive/sexual health consultations. Only one-third knew that midwives can prescribe and administer long-acting reversible contraceptives (LARCs).

**CONCLUSIONS:**

Women want contraceptive consultations to include more information on side effects and available hormonal contraceptives. Women want to be asked about their sexual health and wellbeing during contraceptive consultations. Women should be made aware that midwives can provide contraceptive services including LARCs.

## INTRODUCTION

Reproductive health, sexual wellbeing and the use of contraceptives are closely linked. The United Nations (UN) states that reproductive health implies that people should be able to decide if and when they want to reproduce, and also that their sex life should be safe and satisfying^1^.

The UN reports that 9% of married or in-union women worldwide use contraceptive pills, 14% use intrauterine contraceptive devices (IUDs), and 19% rely on female sterilization^2^. Short-term and reversible methods are more common in Africa and Europe, whereas long-acting and permanent methods are preferred in Asia and Northern America^2^.

Among women of fertile age in Norway, 33% use hormonal contraceptives or non-hormonal IUDs^3^. Approximately half of these women are using combined oral contraceptives (‘the pill’)^4^. The extent of the use of emergency contraceptives such as ‘the morning after pill’ or retrospect insertion of IUDs is not known in Norway. Sale statistics for ‘the morning after pill’ indicate that they add little to the total volume of contraceptives used^5^. There are a number of non-hormonal ways to limit the chances of pregnancy, such as: barrier methods, withdrawal method, breastfeeding, and ‘safe period’ during the menstrual cycle^6^. Sales of barrier devices are not registered and neither is the use of natural family planning methods, nor did we find up-to-date research information on their use in Norway. The Norwegian government, in line with current research, recommends that more women should use Long-Acting Reversible Contraceptives (LARCs)^7,8^. The Pearl index, an index showing the number of unintended pregnancies while using any given contraceptive, is showing that LARCs are the best alternatives to avoid pregnancies. The index is based on how many out of 100 women conceive during a year of both typical and perfect use of a specific contraceptive. Imperfect use is low when the contraceptive is in situ, and not dependent on the user applying or digesting the contraceptive on frequent intervals. As an example, oral contraceptives have a Pearl index as low as 0.3 with perfect use, but as high as 8 with typical use. In comparison, the hormonal IUD has the same typical and perfect use index of 0.1^9^.

Except for the ‘morning after pill’, all hormonal contraceptives are provided through a contraceptive consultation. Easy access to a contraceptive consultation is thus important and essential. One aspect of this consultation is for the provider to ensure there is no predisposition for serious but rare side effects such as deep venous thrombosis, pulmonary thrombosis, myocardial infarction and cerebrovascular accidents^10^. However, less serious but more common side effects such as unplanned pregnancies due to poor compliance, altered bleeding patterns, genital dryness, mood changes, effects on mental health, changes of libido etc. should also be discussed, as they carry great importance with the women who have to live with these effects in their daily life^10^.

There is limited research about Norwegian women’s opinion on the access to and information about contraceptives. However, one large intervention study was conducted by SINTEF (The Foundation for Scientific and Industrial Research at the Norwegian Institute of Technology) among women aged 20–24 years in four Norwegian municipalities in the period 2008–09^11^. In two municipalities, women received free contraception and easy access to care, while women in two other municipalities received standard care. The number of women who used hormonal contraceptives did not differ between the groups^10^. However, in the intervention group there was a significant increase in the proportion of women using LARCs^10^. Concern about side effects, reluctance to add hormones to the body, lack of information on side effects and suitable alternative contraceptives were all major barriers to more women using contraceptives^10^. More recent studies involving all women of fertile age have not been conducted. Since the SINTEF study, the Norwegian government has implemented new guidelines encouraging the increased use of LARCs^7,8^. The effect these implementations have had on women’s experiences and opinions on contraceptive use and contraceptive counselling has not been examined.

Besides the prescription of contraceptives and information about their side effects, the contraceptive consultation offers the opportunity for health care professionals to inquire about women’s sexual health and wellbeing. Neither the SINTEF study nor any other Norwegian study, that we have found, has investigated aspects of sexual health and wellbeing as part of the contraceptive consultation. However, we found one Swedish cross-sectional study by Wendt et al.^12^ of 448 women, which addressed these aspects in a gynecological consultation. This study showed that while few women had been asked, close to 92% of the women considered it appropriate and useful to be asked about sexual health and wellbeing during a gynecological consultation^12^.

The aim of the present study was to investigate Norwegian women’s experiences of and opinions on contraceptive consultations.

## METHODS

### The Norwegian setting

Norwegian youth aged 15–20 years have had access since 2002 to free consultations on sexual health and contraceptives through the public health clinic for youth or school health services. Here they can get prescriptions for free or subsidized contraceptives, including LARCs^13^. This service is offered mainly by public health nurses (PHNs) and some midwives, who have been authorized to prescribe hormonal contraceptives to women aged 16–20 years since 2002^14^. Before this, prescriptions for contraceptives were only available from doctors, with a payable fee both for consultations and contraceptives.

In 2016, the Norwegian government extended the right of midwives and PHNs to prescribe contraceptives to all women of fertile age, not only those 16–20 years of age^8^. This means that women can now choose to see a midwife or a PHN for their contraceptive needs. Most midwives and PHNs work at a public health clinic. Access to a contraceptive consultation has thus, in theory, become easier. However, due to lack of resources, in particularly midwives, most public health clinics still only offer appointments to women under the age of 20 years, and pregnant women, as required by the Norwegian law^13,15^. Other services are offered at each clinic’s discretion.

### Method, sample and design

We conducted a cross-sectional study with a questionnaire distributed via the Internet. Data collection started on the 7 December 2017 and finished 28 February 2018. The survey was distributed with a link to Questback.com on Facebook pages such as sexogsamfunn.no, women’s groups, political organizations, and students’ organizations. Inclusion criteria were women who perceived themselves to be of fertile age, from 15 years of age with no upper age limit defined by the authors.

All authors were involved in the development of the questionnaire. The full questionnaire is available as a supplementary file (Supplementary File 1). It was compiled by questions adapted from the SINTEF survey^11^ regarding access and information given during contraceptive consultations, and questions adapted from the Wendt et al.^12^ study regarding sexual wellbeing. The sociodemographic (independent) variables included were age, employment and size of municipality. To ensure anonymity, age was asked for in categories.

The second section was about access and information on contraceptives. Women were asked how many times they had been to a consultation regarding contraceptives the previous year: none, 1–2 or ≥3. Participants currently not using hormonal contraceptives were asked if a different access would make them consider using them. All participants were asked how easy or difficult they found it to access consultations. Only women who had been to consultations regarding hormonal contraceptives were asked what topics they wanted more information on. If the participants had not used hormonal contraceptives in the last six months, they were asked why not. This question had 12 answering options and the women could tick off all relevant ones. In addition, women were asked if they had an unplanned pregnancy during the last year.

The third section included questions on sexual wellbeing. Women were asked if questions on sexual wellbeing had been raised during contraceptive consultations they had attended, and whether they deemed the topic important or not.

In the last section of the questionnaire participants were asked if they would find it appropriate to see a midwife regarding questions on contraceptives, sexual health and wellbeing, and if they were aware that midwives can administer IUDs and contraceptive implants. Finally, there was a section for free comments.

### Data analysis

We performed descriptive statistical analysis; frequencies and proportions as well as cross-tabulation. The Statistical Package for Social Sciences (SPSS) version 24 for Windows was used to perform the analysis. Bivariate analysis was also performed, using a Pearson chi-squared test with 95% confidence intervals. There were little missing data, and missing values were not replaced.

The cross tabulations by municipality and employment status showed no significance regarding the dependent variables. Therefore, bivariate analyses presented in the tables are performed with age groups and the dependent variables only.

For analysis, age was recoded into three age groups: 15–24, 25–34 and ≥35 years. Answers to the question considering consultation access were recoded to binary results showing how many women found it hard to access consultations. Similarly, on whether women would use hormonal contraceptives if the service was different, answers were dichotomized to ‘yes’ with all other answers becoming ‘no’.

### Ethics

NSD, Norwegian Centre for Research data, was consulted for initial approval, but application was not needed as total anonymity was ensured^16^. Information about the purpose of the survey and how the anonymity of the respondent was secured were stated in the introduction of the questionnaire (Supplementary File 1). Responding to the questionnaire was considered consenting to the use of the gathered data. We considered the possibility that painful emotions could be provoked due to the intimate nature of some of the questions. However, this was outweighed by the likely positive effect that women could become more aware that they can raise such issues next time they seek contraceptive counselling. The participants IP addresses were not accessible at any stage during the research. The data were stored at Questback.com and the results were sent by email as an SPSS data file.

## RESULTS

A total of 1917 women responded to the questionnaire. Of all the women participating, 67% were under 35 years of age and 24% were students (Table 1). Among the age group 15–24 years, 63% were currently undertaking studies. Of all participants, 53% reported that they had been using hormonal contraceptives the last six months and 6% of the women had had an unplanned pregnancy the last year (Table 1).

**Table 1:** Characteristics of the participants (N=1917)

*Variable*	*n*	*(%)*
**Age**		
15–24	397	(20.7)
25–34	890	(46.4)
≥35 years	630	(32.9)
**Size of municipality by inhabitants**		
>30000	1193	(62.3)
10000–30000	451	(23.6)
<10000	271	(14.2)
Missing	2	(0.1)
**Employment status**		
Employed	1289	(67.4)
Student	459	(23.9)
Unemployed	48	(2.5)
Other	116	(6.1)
Missing	5	(0.2)
**Using hormonal contraceptives last 6 months**	1028	(53.6)
**Unintended pregnancies last year**	117	(6.1)

The youngest women (15–24 years) were the most frequent users of contraceptive consultations, with 63% seeing a health care professional at least once during the last year, compared to 33% of those ≥35 years (Table 2). Almost none of the women found it hard to get an appointment for a contraceptive consultation (Table 2).

**Table 2:** Frequency of and access to contraceptive consultations by age groups^a^ (N=1917)

*Item*	*Age group*						*Total*		*p^b^*
	*15–24 n (%)*		*25–34 n (%)*		*≥35 years n (%)*		*n (%)*		
**Consultations last year (n=1910)**									
None	144	(36.5)	488	(55.0)	423	(67.5)	1055	(55.2)	<0.001
1–2	218	(55.2)	372	(41.9)	196	(31.3)	786	(41.0)	<0.001
≥3	33	(8.4)	28	(3.2)	8	(1.3)	69	(3.6)	<0.001
Missing	2	(0.5)	2	(0.2)	3	(0.5)	7	(0.03)	
**Access to consultations (n=1869)**									
Find it hard to access	21	(5.3)	46	(5.2)	29	(4.8)	96	(5.0)	0.877
Easy/Neither easy nor hard	366	(94.6)	828	(94.7)	579	(95.2)	1773	(94.9)	
Missing	2	(0.5)	2	(0.2)	3	(0.5)	7	(0.03)	

a Column percentages

b Chi-squared test for significance

The majority of women across all age groups wanted more information about side effects, with no significant differences between the age groups. The women also expressed a need for more information on alternative available contraceptives. This was significantly more important for the women aged 15–34 years compared to those ≥35 years (Table 3).

**Table 3:** Wish for more information by women who had ever used hormonal contraceptives^a^ (N=1477)

*Item*	*Age group*						*Total*		*p^b^*
	*15–24 n (%)*		*25–34 n (%)*		*≥35 years n (%)*		*n (%)*		
Available contraceptives	172	(50.7)	383	(54.2)	169	(39.2)	724	(49.0)	<0.001
Side effects	239	(70.5)	467	(70.3)	283	(65.7)	1019	(69.0)	0.206
General information	196	(57.8)	317	(44.8)	170	(39.4)	683	(46.2)	<0.001
Other subjects	37	(10.9)	59	(8.3)	44	(10.2)	140	(9.5)	0.343

a Multiple choices available.

b Chi-squared test for significance.

The most common reason for not using hormonal contraceptives was concern about the side effects (Supplementary File 2).

There were significant differences between the age groups regarding which topics of sexual health and wellbeing were deemed important to be raised in a contraceptives was concern about the side effects contraceptive consultation (Table 4). For example, control over one’s sexuality and/or pleasure in sexual relationships is rated considerably more important by women less than 35 years compared to women 35 years and older (p<0.001). Significant discrepancy was observed between whether the topics had been raised during the consultation or the women found it important that the topics were raised (Table 4). This applies to all the questions on sexual health and wellbeing (Table 4).

**Table 4:** Women’s opinions on questions regarding sexual health and wellbeing during contraceptive consultations (N=1829)

*Item*		*Age group*						*Total*		*p^b^*
		*15–24 n (%)*		*25–34 n (%)*		*≥35 years n (%)*		*n (%)*		
‘How are you?’	Subject raised	156	(41.1)	313	(36.7)	222	(37.3)	691	(37.8)	0.325
	Deemed important	306	(78.3)	677	(77.3)	451	(73.8)	1434	(76.4)	0.184
Control/pleasure in sexual relations	Subject raised	82	(21.8)	125	(14.7)	78	(13.3)	285	(15.7)	<0.001
	Deemed important	279	(71.7)	572	(65.8)	348	(57.2)	1199	(64.3)	<0.001
Body image/self-esteem	Subject raised	38	(10.1)	84	(9.8)	56	(9.7)	178	(9.8)	0.977
	Deemed important	256	(66.0)	564	(64.7)	345	(56.7)	1165	(62.3)	<0.001
Genital discomfort or pain	Subject raised	89	(23.5)	190	(22.2)	164	(28.1)	443	(24.4)	0.036
	Deemed important	348	(89.5)	811	(92.9)	554	(91.0)	1713	(91.6)	0.105
Feeling of guilt or shame	Subject raised	35	(9.2)	60	(7.1)	33	(5.6)	128	(7.1)	0.107
	Deemed important	290	(74.7)	609	(70.2)	373	(61.6)	1272	(68.4)	<0.001
Sexual abuse and/or violence	Subject raised	55	(14.4)	77	(9.1)	51	(8.7)	183	(10.1)	0.006
	Deemed important	339	(87.8)	731	(84.3)	469	(77.4)	1539	(82.2)	<0.001

a Chi-squared test for significance.

Close to half of the women found it appropriate to see a midwife for contraceptive counselling or about issues concerning sexuality (Table 5). One-third knew that midwives could administer LARCs. There were no significant differences between the age groups (Table 5).

**Table 5:** Midwives as counsellors on contraceptive and sexual health issues (N=1902)

*Item*	*Age group*						*Total*		*p^b^*
	*15–24 n (%)*		*25–34 n (%)*		*≥35 years n (%)*		*n (%)*		
‘Would you consider it appropriate to see a midwife about contraceptive/sexual health needs?’	172	(43.5)	410	(46.4)	303	(48.6)	885	(46.5)	0.282
‘Did you know midwives can prescribe and administer LARC?’	134	(33.8)	295	(33.4)	225	(36.1)	654	(34.4)	0.535

b Chi-squared test for significance.

## DISCUSSION

Very few women found access to contraceptive consultations to be an issue. Over two-thirds of all women wanted more information on side effects when attending contraceptive consultations. Insufficient information was also the main reason for non-use of hormonal contraceptives. All the different aspects of sexual wellbeing were deemed important to talk about during contraceptive consultations by over 50% of the women across all age groups. The women also indicated that the sexual wellbeing aspect of contraceptive care was under-communicated during these consultations. Almost half of the women found it appropriate to see a midwife for contraceptive/sexual health counselling. Only one-third knew that midwives can prescribe and administer long-acting reversible contraceptives (LARCs).

Very few women in our survey found it hard to get an appointment for contraceptive consultation. The access to contraceptive consultations in Norway differs slightly from other comparable European countries. In the United Kingdom contraceptives are available from most general practices, specialist community contraception clinics, sexual health clinics, some genitourinary medicine clinics and pharmacies^17^. Swedish women mainly see midwives in community health clinics^18^ where they also get general care for their health and wellbeing. One could argue that the Norwegian setting, as described earlier, puts more limits on women’s access to contraceptive consultations. However, the women included in our study reported very little difficulty regarding access to contraceptive consultations.

Across all age groups, women wanted more information about the side effects of contraceptives. The youngest women expressed a significantly higher need for general information on contraceptives compared to the other age groups. This increased need for general information among the youngest participants could be due to their lack of experience with contraceptive use, compared to the women of older age. Still, the youngest women in our study expressed a higher need for general information than the women in the SINTEF study where 42% of women wanted more general information^11^. The difference might be explained by increased focus in media on side effects, rising incidence of sexually transmitted infections^19^, and more focus on LARCs since the time of the SINTEF study.

In our study, 53% of the participants had not used hormonal contraceptives the past year, slightly less than in the Norwegian national statistics^3^. The difference could be due to the fact that our study included a higher proportion of younger women. Concerns about side effects were given as the main specific reason for non-use in all age groups. This is in line with findings from other, international studies, which looked at reasons for contraceptive non-use among women^20-22^. Considering heavy news coverage of recent research on hormonal contraceptives increasing the incidence of depression and suicide^23,24^, it is reasonable to believe that women can be reluctant to use them. Women need to receive information on the rare but serious side effects of contraceptives. In addition, other more common but less serious side effects such as irregular bleeding, weight gain, mood changes, changes in libido etc. should be discussed with the women’s personal preferences and needs in mind. Bitzer et al.^25^ suggest a method of personalized interactive consultations that allow these individual needs to be addressed and also for the choice of contraceptive to be assessed and re-evaluated with regard to its effect on a woman’s biopsychosocial life^25^.

In our sample, only a small proportion of the women had been asked questions on sexual wellbeing during contraceptive counselling, while a high proportion thought it to be important. The subjects of genital discomfort or pain, and history of sexual abuse or sexual violence, stood out as the topics that women of all age groups found most important to address in these consultations. Yet, very few had been asked about these issues. Compared to the findings of Wendt et al.^12^, women in our study were asked about these issues more often. There has been an increasing focus on sexual violence and abuse both internationally and in Norway over the past few years, with a dedicated WHO directive^26^ and Norwegian governmental guidelines^27^. This might be an explanation for the increase. Unorganized movements such as #metoo are also currently raising awareness around women who have been subject to unwanted sexual attentions and abuse. This too might affect the views that women have on talking about their own experiences with a health care professional. The subject of ever having experienced sexual abuse or violence was broached with the women of the youngest age group more frequently than with the other groups, but rated as equally important by all women aged 15–34 years. Almost one in ten women in Norway has been subject to rape, and the likelihood of having experienced sexual abuse increases with every year lived^28^, making this a relevant subject across all age groups.

It can be difficult for women to find a person they can confide in when experiencing problems with shame, body image, genital pain, control over their sexuality or similar issues. However, over half of the women across all age groups found all the subjects raised in the questionnaire important to talk about in a contraceptive consultation. It is easier to answer directly asked questions than to bring up subjects oneself. Therefore, it would be appropriate if health care personnel ask questions and invite open dialogue about the wider perspective on sexual health and wellbeing^29^. Contraceptive consultations offer an opportunity to discuss these issues.

All women rated vaginal pain and discomfort important to talk about, but women 35 years and older were asked significantly more often. This might indicate that health care personnel consider it more appropriate to talk about this with women who have gone through childbirth with the accompanying trauma to the tissues. Similarly, women are more likely to start developing menopausal symptoms such as vaginal dryness and thinning of the mucous membranes after the age of 35 years. Younger and/or nulliparous women might be considered less likely to experience genital pain. However, the presumption that questions about vaginal pain and discomfort are more appropriate with increasing age is not accurate. Seventy-five per cent of all women who have vulvodynia (vaginismus, vulvar vestibulitis and generalized vulvar pain) experience the onset before the age of 34 years^30^, and up to 16% of women experience this pain syndrome^31^.

Close to half of the women across all age groups considered midwives appropriate health care personnel to consult when they have questions about contraceptives or need advice on sexual health. We had expected fewer among the youngest women to give this answer, as less of them have yet met the midwife through community antenatal care. Only about one-third of the women were aware that midwives can prescribe and administer LARCs. This shows that there is a lack of knowledge among women about what services midwives can provide. Besides midwives being given the authority to prescribe, women need to be informed about their options and sufficient midwives need to be employed at community health centers.

### Limitations and strengths

The questionnaire was electronic and spread via the internet, mainly through Facebook, so women needed access to the internet, a computer, and in many instances also a Facebook account, to participate. The questionnaire was in Norwegian only, requiring the women to being able to read and comprehend the language. This could lead to selection bias; thus our sample may not be representative for the Norwegian population in general. We did not ask about ethnicity and therefore do not know how many nonnative Norwegian speakers participated.

Overrepresented in our sample are women 20–39 years of age, with less respondents from age groups 15–19 and >45 years. This might be because the youngest women frequent Facebook less than other social media platforms, and that the older women felt the questionnaire did not apply to them. As the results are presented by age groups, it is easy to interpret the findings for the different age groups without generalizing to all women. It is uncertain whether anyone chose to answer multiple times, or if in fact all respondents were female.

A strength of our study is that we have a large sample. Another strength is that we included questions previously used by experts in the field^11,12^, even though a couple of questions were modified.

A weakness of our study is that the results are descriptive only, and no multivariate regression analyses were performed. However, these analyses should be used with caution in a cross-sectional study.

## CONCLUSIONS

Access to contraceptive consultations was not a significant obstacle to contraceptive use. Women want consultations to include significantly more information on side effects and available alternative hormonal contraceptives. They also want information and communication about general sexual wellbeing such as vaginal pain and discomfort, and history of sexual violence and abuse. Just under half of the women view midwives as appropriate health care professionals to see on these matters.

More focus needs to be placed on meeting the woman’s individual needs during the contraceptive consultation. Not only on the right type of contraceptive, but also her general sexual health and wellbeing. Women need to be made aware that midwives can provide contraceptive services, and midwives should consider initiating political action to be able to use their right to prescribe in the community.
