# Supplementary material for: Contraceptive consultations: A cross-sectional study of Norwegian women's experiences and opinions
Source: Eur J Midwifery. 2019 Jun 26;3:12. doi: 10.18332/ejm/109773 (PMC7839094; doi:10.18332/ejm/109773)
Supplement: Supplementary file 1 [file EJM-3-12-s1.pdf]

Supplement 2. Reason for not using hormonal contraceptives the last 6 months (N = 1028).

	Age 15-24 *n (%)*		Age 25-34 years *n (%)*		Age <35 *n (%)*		Age Total *n (%)*		p-value
Not sexually active	35	(24.0)	41	(8.1)	49	(13.0)	125	(12.2)	< .001
Planning pregnancy	16	(11.0)	139	(27.5)	47	(12.5)	202	(19.6)	< .001
Female partner	7	(4.8)	17	(3.4)	9	(2.4)	33	(3.2)	.364
Scared/worried about side-effects	53	(36.3)	153	(30.2)	93	(24.7)	299	(29.1)	.024
Consider contraceptives too expensive	12	(8.2)	17	(3.4)	4	(1.1)	33	(3.2)	< .001
Using withdrawal method, safe periodes, condom	40	(27.4)	111	(21.9)	60	(16.0)	211	(20.5)	.008
Against exposing the body to hormones	32	(21.9)	133	(26.3)	101	(26.9)	266	(25.9)	.490
Recently moved, unsure where to get prescription	4	(2.7)	1	(0.2)	0	(0.0)	5	(0.5)	< .001
Difficult to get appointment that suits me	9	(6.2)	7	(1.4)	1	(0.3)	17	(1.7)	< .001
Unsure which contraceptives suits me	23	(15.8)	51	(10.1)	17	(4.5)	91	(8.9)	< .001
Reluctant to contact health personnel	9	(6.2)	14	(2.8)	5	(1.3)	28	(2.7)	.010
Other reasons	31	(21.2)	132	(26.1)	153	(40.7)	316	(30.7)	< .001

© 2019 Forsberg K. E.
