# Supplementary material for: Contraceptive consultations: A cross-sectional study of Norwegian women's experiences and opinions
Source: Eur J Midwifery. 2019 Jun 26;3:12. doi: 10.18332/ejm/109773 (PMC7839094; doi:10.18332/ejm/109773)
Supplement: Supplementary file 2 [file EJM-3-12-s2.pdf]

Supplement 1
Questionnaire

This questionnaire was originally distributed in Norwegian only, and the translation to English has been done by the first authors of the article for the purpose of publication.

1) **How old are you?**

15-19
20-24
25-29
30-34
35-39
40-44
45 or older

2) **Currently, are you?**

Employed
Student
Not working/unemployed
Other

3) **Where do you live?**

Large municipality (over 30 000 inhabitants)
Medium sized municipality (over 10 000 inhabitants)
Small municipality (less than 10 000 inhabitants

4) **Have you been to see a doctor/community nurse/midwife for questions/needs regarding contraceptives or to get a prescription for contraceptives during the last year?**

No
Yes, 1-2 times
Yes, 3 or more times

Below follow a few questions on hormonal contraceptives. These include oral contraceptives, hormonal intrauterine devices, contraceptive injections, transdermal contraceptive patches, contraceptive vaginal ring and subdermal contraceptive implants.

5) **If you have NOT used hormonal contraceptives during the last six months, what is the reason for this? You can tick of multiple reasons**

I'm currently not sexually active
I wish to become pregnant
I have a female partner
I'm worried about side effects
It's too expensive
I use barrier methods/withdrawal method/safe periods
I oppose to adding hormones to my body
I've relocated and don't know where to get my prescriptions
It's hard to get appointments for contraceptive counselling at a time that suits me
I'm unsure as to what hormonal contraceptive that would be suitable to me
I'm uneasy about contacting/interacting with health care personnel
Other reason

6) **Would you consider using hormonal contraceptives if you could:**

(*Note to reader of article: Answering options in the online questionnaire were "Yes", "No","Don't know/doesn't matter")*
Get appointment with a midwife/PHN at a Community health centre
Get appointment free of charge
Get appointment within a week
Get appointment after office hours

7) **If hormonal contraceptives have been prescribed to you, did you wish for more information regarding (multiple options available):**

Alternative contraceptives/different options
Side effects
Emergency contraceptives
Other subjects

8) **How easy or hard do you find it to access a contraceptive consultation?**

Easy

Neither easy or hard
Hard

9) **Have you had an unplanned pregnancy the last year?**
Yes
No

Below follow a few topics we would like to know whether they were discussed or not when you attended a contraceptive consultation. We would also like to know if you consider these topics important to discuss during a contraceptive consultation. (If you haven't been to a contraceptive consultation, answer only if you consider the topics important to discuss in such a setting).

10) **Where these subjects raised , options "Yes"/"No"**
How are you?
Do experience control in/enjoy your sexual relations?
How is your body image, self-consciousness?
If you experience genital discomfort/pain during sexual actions or in general?
If you experience guilt/shame in regards to your sexuality/in your sexual relations (i.e. in relation to cultural/religious norms, parental expectations)?
If you have experienced abuse/sexual violence?

11) **How important is it that that these topics are raised?**
(*Note to reader of article: Answering options in the online formula were "Not important"/ "Of little importance"/ "Quite important"/"important"/ "Very important")*
How are you?
Do experience control in/enjoy your sexual relations?
What is your body image, self-consciousness?
If you experience genital discomfort/pain in your sexual relations or in general?
If you experience quilt/shame considering your sexuality/in your sexual relations (because of for example cultural challenges, parental expectations)?
If you have experienced abuse/sexual violence?

12) Midwives are often associated with pregnancy and birth, but they also have competence and knowledge about sexual health and sexuality. They can prescribe contraceptives to woman of all ages.
Would you find it natural to see a midwife considering your needs in these areas?
Yes
No

13) Did you know that midwives can insert IUD's and subdermal contraceptive implants?
Yes
No

14) Are there any other thoughts or issues you consider important regarding health personnel, contraceptive consultation, contraceptives and sexuality/sexual wellbeing you want to share with us? Leave a comment!

© 2019 Forsberg K. E.
